# Adenocarcinoma developing from gastric heterotopic pancreas: a case report and short review

**DOI:** 10.3389/fsurg.2024.1274389

**Published:** 2024-05-09

**Authors:** Ran Qi, Kun Li, Baomin Shi

**Affiliations:** ^1^ Department of General Surgery, Xinhua Hospital, School of Medicine, Shanghai Jiao Tong University, Shanghai, China; ^2^ Department of Hepatobiliary Surgery, The First Affiliated Hospital of Dalian Medical University, Dalian Medical University, Dalian, China

**Keywords:** ectopic pancreas, heterotopic pancreas, adenocarcinoma, obstructive jaundice, submucosal gastric lesions

## Abstract

Heterotopic pancreas is a relatively rare condition that may be associated to clinical complaints or signs. Here, we report a case of gastric heterotopic pancreas assictaed to ductal adenocarcinoma. Obstructive jaundice was the initial symptom prompting medical intervention. A 73-year-old male patient presented with yellow staining of the skin and sclera, and dull epigastric pain. Contrast-enhanced computed tomography showed stenosis of the extrahepatic distal bile duct and mass lesions of the antrum. The patient underwent tumor resection, distal gastrectomy (Billroth II), and common bile duct exploration. Postoperative pathological examination revealed an adenocarcinoma located in the wall of the gastric antrum. Immunohistochemical results suggested that the tumor originated from the pancreas. Heterologous pancreatic tissue and a dilated pancreatic duct were found in the tumor. These findings suggest malignant transformation of the gastric heterotopic pancreas. Of note, jaundice as clinical complaint for adenocarcinoma associated to gastric heterotopic pancreas.

## Introduction

Heterotopic pancreas is a congenital abnormality that occurs mostly in the gastrointestinal tract, but also in the liver, thyroid, middle ear, and other areas (1). Most heterotopic pancreatic of the upper gastrointestinal tract were found out in the antral wall. Heterotopic pancreas has been reported to have a frequency of 1/500 in surgical specimens (2). Although heterotopic pancreas often has no clinical symptoms, pathological changes such as bleeding (3), pancreatitis (4), and malignant transformation (5) occur. Due to the absence of typical symptoms, malignant transformation of heterotopic pancreas is difficult to detect and diagnose, and thus, heterotopic pancreas cancer is associated with a poor prognosis (6). This report contains the description of a case of malignant heterotopic pancreas where obstructive jaundice was the initial symptom. Although the tumor was successfully surgically removed, the patient ultimately died of sepsis and multiple-organ failure 3 weeks after surgery.

## Case report

A 73-year-old male patient was admitted to the hospital complaining of dull epigastric pain and yellow staining of the skin and sclera for 2 weeks. The patient presented without chills, was afebrile, and reported normal bowel movements. The patient denied a history of smoking and excessive alcohol consumption. A physical examination on admission revealed epigastric abdominal tenderness, no palpable masses, and negative Murphy sign. Laboratory results indicated abnormal liver function; ALT: 194 (9–50 U/L), AST: 136 (15–40 U/L), and T-BIL: 112 (5.1–20.5 μmol/L). Blood tumor marker tests showed elevated carbohydrate antigen 19-9: 79.68 (<39 U/ml) and carbohydrate antigen 50: 34.79 (<25 U/ml). There was no obvious abnormality in their complete blood count.

**Figure 1:**
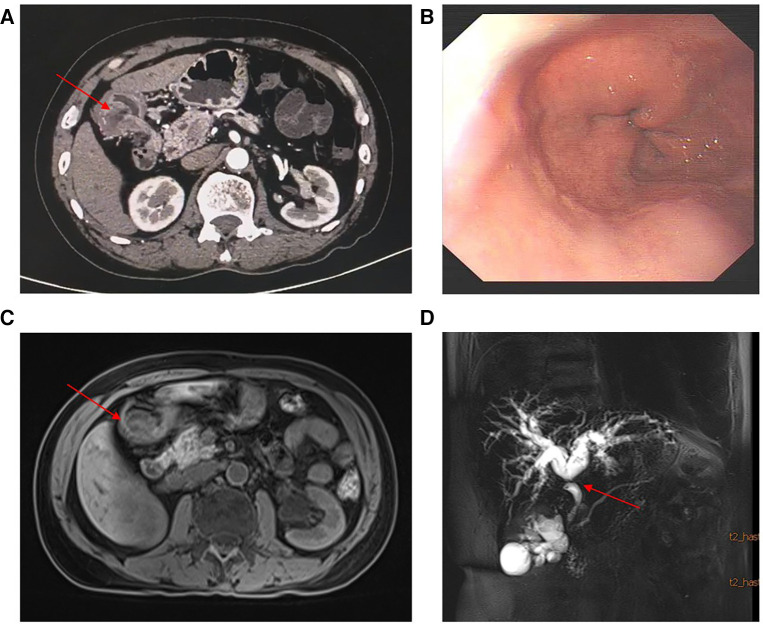
(**A**) Contrast-enhanced computed tomography showed stenosis in the extrahepatic distal bile duct and a mass with nodular enhancement in the gastric antrum. (**B**) Gastroscopy showed pyloric stenosis and no mucosal protrusion. (**C**–**D**) Enhanced nuclear magnetic resonance and MRCP showed the mass and dilatation of the bile duct and local stenosis of the extrahepatic distal bile duct.

Images obtained by computed tomography scan after contrast-substance-injection showed stenosis in the extrahepatic distal bile duct and thickening of the gastric wall in the antrum with nodular enhancement. A mass measuring about 3 × 3 × 2 cm was seen on the dorsal side of the antral wall. No suspicious lesions were found elsewhere in the abdomen. Magnetic resonance cholangiopancreatography (MRCP) showed dilatation of the bile duct and confirmed a stenotic extrahepatic distal bile duct. No significant abnormalities were found in pancreatic duct. No pancreatic abnormalities or heterotopia were found. Gastroscopy showed pyloric stenosis without mucosal protrusion or erosion (Figure 1). These laboratory and imaging studies indicated the presence of a malignant tumor and a preliminary diagnosis of cholangiocarcinoma.

**Figure 2:**
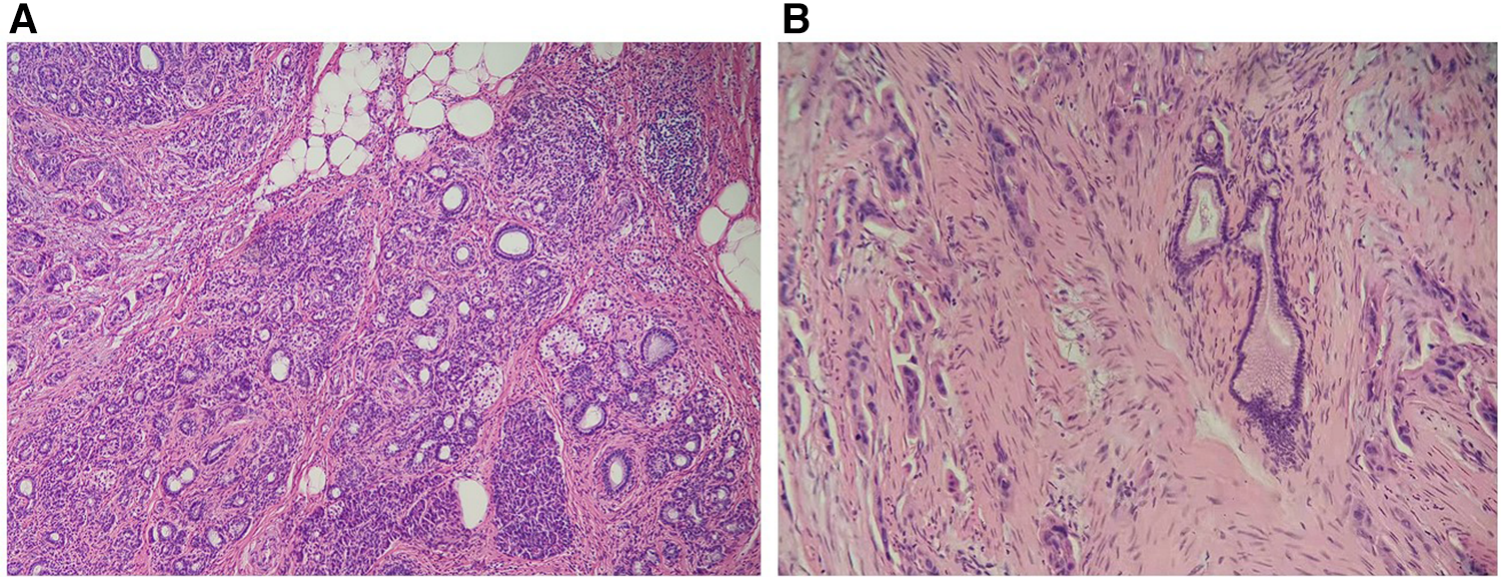
(**A**) Heterotopic pancreas tissue in the wall of the stomach, with acinar ducts visible. (×100 magnification). (**B**) Dilated pancreatic ducts and moderately atypical adenocarcinoma cells can be seen in the heterotopic pancreas (×200 magnification).

During surgery, a 3.2 × 3.6 × 3.3 cm tumor was located at the proximal end of the antrum duodenal bulb, anterolateral to the common bile duct. Rapid intraoperative frozen pathology identified the mass as an adenocarcinoma. Tumor resection, distal gastrectomy (Billroth II), and common bile duct exploration were subsequently performed. Postoperative pathological examination showed that the lesion contained heterogenous pancreatic and gastric wall tissue with dilated pancreatic ducts. The tumor invaded the muscular layer of the gastric wall without reaching the submucosa. There was the presence of vascular emboli in the tumor and without perineural tumor invasion. A large number of atypical cells of pancreatic origin were found and identified as being closely associated with heterotopic pancreatic tissue (Figure 2). The heterotopic pancreas was completely surrounded by adenocarcinoma. Immunohistochemical studies: NSE(-), CgA(+), CD56(NK-1)(+). The tumor metastasized to the third lymph node. The tumor was therefore diagnosed as adenocarcinoma arising from gastric heterotopic pancreas. Two weeks after surgery, the patient developed postoperative abdominal effusion with infection, hepatic insufficiency, and hypotension, and he was subsequently transferred to the ICU for treatment. Three weeks following the surgery, the patient developed a severe infection resulting in multiple-organ failure, and the patient eventually died.

## Discussion

Heterotopic pancreas, also known as an aberrant or ectopic pancreas, is defined as pancreatic tissue in an abnormal location without any anatomical, vascular, or neural continuity with the normal pancreas. Heterotopic pancreas is a congenital and developmental anomaly arising during embryogenesis. According to Heinrich (1909) and Gaspar-Fuentes (1973), heterotopic pancreas is organized into the following histological classifications: Type I, consisting of ducts, acinar cells, and Langerhans islets; Type II, containing pancreatic ducts only; Type III, composed of primarily of acinar tissue; and Type IV, comprised only of islet cells (1, 7). This case reports on an ectopic pancreas of the type I variety because all of the requisite elements were present. Patients with heterotopic pancreas are usually asymptomatic, so the anomaly is often only discovered through routine imaging or endoscopy, after surgery, or even post-mortem on autopsy. The incidence of heterotopic pancreas reported during autopsy was 0.55%–13.7%, while those reported following upper abdominal surgery was 0.2% (8). However, because of the lack of large-scale statistics, the true incidence of heterotopic pancreas may be higher. Heterotopic pancreas is typically a benign disease that does not require aggressive surgical resection. Although heterotopic pancreas may result in bleeding, pancreatitis, intraductal papillary mucinous neoplasm (IPMN) (9), and other lesions, heterotopic pancreas is associated with a favorable prognosis in most patients. Malignant transformation of heterotopic pancreas is considered a rare event, with a reported incidence of 0.7%–1.8% (10). Where heterotopic pancreatic adenocarcinoma is concernced, three distinct diagnostic criteria have been proposed. First, the tumor must be in or near the ectopic pancreas. Second, the transition between pancreatic tissue and tumor should be established. Third, non-neoplastic pancreatic tissue should contain well-developed ducts and acinus (11).

The diagnosis of malignant transformation of heterotopic pancreas tissue is challenging because the clinical symptoms and imaging features of these tumors are not well defined. As with many cancers, the malignant heterotopic pancreas is often asymptomatic in its early stages, and the symptoms experienced during middle and late stages depend heavily upon the dysfunction of the infiltrated organ and the resultant external pressure exerted on that organ. Laboratory tests for CA19-9 and carcinomaembryonic antigen (CEA) in this case were helpful in diagnosing heterotopic pancreatic cancer, although the good specificity of tests for CA19-9 and CEA or other carbohydrate antigen markers are not well established in the medical literature. Diagnosing heterotopic pancreas using conventional imaging studies such as CT and ultrasound is difficult because they only show the presence of a mass but do not reveal any additional defining features. Because heterotopic pancreatic tissue is usually located in the submucosa or muscular layer, upper endoscopy can reveal a protuberant lesion, but mucosal biopsy often fails to obtain the target tissue. Several reported cases have shown that endoscopic ultrasound-guided fine needle aspiration is a reliable method for the diagnosis of ectopic pancreatic adenocarcinoma (12, 13). However, Cazacu et al. reported surgical resection as the predominantly successful diagnostic method for malignant heterotopic pancreas (6). In this case, we did not perform endoscopic ultrasonography because the tumor was considered to be in the serosal layer and the patient needed surgery as soon as possible.

Although survival appears to be superior to primary pancreatic ductal adenocarcinoma (PDAC), the overall prognosis for malignant heterotopic pancreas is poor. As of December 2022, there were 73 cases of malignant heterotopic pancreas published in English literature in the PubMed and Web of Science databases (Table 1).

**Table 1:** Clinical features and follow-up of malignant heterotopic pancreas.

Year of report	Gender/Age	Location	Heinrich typ	Follow-up/Outcome
1962	F/55	Stomach	Ⅰ	6 years/Recurrence
1979	F/55	Stomach	Ⅱ	1 month/Death
1980	M/54	Stomach	Ⅱ	9 months/Death
1980	F/28	Stomach	Ⅰ	2 years/Survival
1990	F/13	Colon	Ⅰ	3 years/Survival
1991	M/42	Stomach	Ⅱ	4 months/Survival
1994	M/60	Hiatus hernia	Ⅰ	11 months/Survival
1996	M/45	Esophagus	Ⅰ	3 years/Survival
1998	F/21	Spleen	Ⅰ	4 years/Death
1999	M/71	Jejunum	Ⅰ	1 years/Metastasis
2001	F/57	Stomach	Ⅱ	13 months/Death
2001	M/60	Stomach	Ⅰ	4 months/Death
2002	F/69	Spleen	Ⅰ	1 year/Survival
2002	M/64	Stomach	Ⅰ	1years Survival
2003	M/64	Duodenum	Ⅰ	10 months/Death
2004	M/52	Stomach	Ⅲ	9 months/Survival
2004	M/85	Stomach	Ⅰ	1 month/Survival
2005	F/74	Ligament of Treitz	Ⅰ	4 months/Survival
2005	F/58	Stomach	Ⅱ	1.5 years/Survival
2006	M/72	Duodenum	Nd	16 months/Death
2007	F/11	Brain	Ⅰ	2 years/Recurrence
2008	F/64	Jejunum	Ⅰ	5 months/Death
2009	M/50	Meckel's diverticulum	Ⅳ	1.5months/Death
2010	F/42	Rectum	Ⅰ	5 years Recurrence
2011	M/3	Omentum	Ⅰ	1 year/Survival
2012	F/66	Anterior mediastinum	Ⅰ	15 months/Death
2012	F/75	Stomach	Ⅰ	11 years/Survival
2012	F/62	Duodenum	Ⅰ	12 months/Survival
2014	M/75	Duodenum	Ⅰ	5 years/Survival
2014	M/73	Stomach	Ⅰ	2 years/Metastasis
2015	M/64	Duodenum	Ⅳ	33 months/Death
2015	F/65	Jejunum	Ⅱ	9 months/Survival
2016	M/76	Ileum	Ⅱ	9 months/Death
2016	M/47	Liver	Ⅲ	56 months/Survival
2017	M/45	Stomach	Ⅱ	12 months/Survival
2018 (14)	M/58	Meckel diverticulum	Ⅰ	4 years/Survival
2018 (15)	F/31	Liver	Ⅳ	4 years/Survival
2019 (16)	F/81	Jejunum	Ⅱ	12 months/Survival
2019 (17)	M/81	Jejunum	Ⅰ	18 months/Survival
2020 (18)	M/74	Colon	Ⅰ	3 years/Survival
2020	F/78	Colon	Ⅰ	3 years/Survival
2020 (19)	F/44	Stomach	Ⅲ	12 months/Survival
2020 (20)	M/77	Duodenum	Ⅰ	9 months/Survival
2020 (21)	M/75	Stomach	Nd	6 months/Survival
2020 (22)	F/70	Duodenum	Nd	10 months/Survival
2021 (23)	F/65	Duodenum	Ⅰ	24 months/Recurrence
2021 (9)	F/71	Stomach	Ⅰ	8 months/Survival
2022 (24)	F/60	Esophagus	Ⅲ	57 months/Death
2022 (25)	M/69	Stomach	Nd	4 years/Recurrence

Nd: No data; F: female; M: male. Data from 1962 to 2017 comes from Cazacu et al. (6).

Prognosis and follow-up records were available in 49 cases. According to these disclosed follow-up data the mortality rate after diagnosis of malignant heterotopic pancreas was 14.3% (7/49) within 1 year and 24.5% (12/49) within 3 years. Recent data show a 5-year overall survival rate of 11% for pancreatic ductal adenocarcinoma (26). Our statistical results showed that compared with primary PDAC, the short-term prognosis of malignant heterotopic pancreas seems to be better. Our study provides the most complete and updated statistical review on the prognosis of malignant heterotopic pancreas; however, the accuracy of our results is limited to the incomplete data available.

Guidelines for the clinical management of heterotopic pancreas have not been established, but available reports suggest surveillance of the dynamics of an ectopic pancreas after examination is warranted. Although postoperative chemotherapy has been reported in patients with malignant heterotopic pancreas, there is no evidence that chemotherapy can significantly improve the prognosis of malignant heterotopic pancreas (27). Radical surgery is the only reliable treatment option at present.

In conclusion, our case report suggests that obstructive jaundice may be the first objective symptom of malignant heterotopic pancreas and that heterotopic pancreas should be considered as a source of malignancy.

## Data Availability

The original contributions presented in the study are included in the article/Supplementary Material, further inquiries can be directed to the corresponding author.
